# Di­aqua­bis­[5-(pyrazin-2-yl-κ*N* ^1^)-3-(pyridin-3-yl)-1,2,4-triazolido-κ*N* ^1^]zinc

**DOI:** 10.1107/S1600536814004176

**Published:** 2014-03-05

**Authors:** Ye-Nan Wang, Wen-Wen Dong

**Affiliations:** a College of Materials & Chemical Engineering, China Three Gorges University, Yichang 443002, People’s Republic of China

## Abstract

In the title compound, [Zn(C_11_H_7_N_6_)_2_(H_2_O)_2_], the Zn^II^ cation, located on an inversion center, is *N*,*N*′-chelated by two 5-(pyrazin-2-yl)-3-(pyridin-3-yl)-1,2,4-triazolide anions and is further coordinated by two water mol­ecules in a distorted N_4_O_2_ octa­hedral geometry. In the anionic ligand, the pyrazine and pyridine rings are twisted with respect to the central triazole ring by 5.77 (10) and 11.54 (10)°, respectively. In the crystal, classical O—H⋯N and weak C—H⋯O hydrogen bonds and π–π stacking inter­actions between aromatic rings [the centroid–centroid distances between triazole and pyrazine rings, and between triazole and pyridine rings are 3.623 (2) and 3.852 (2) Å, respectively] connect the mol­ecules into a three-dimensional supra­molecular architecture.

## Related literature   

For applications and related structures of 1,2,4-triazole derivatives, see: Zhang *et al.* (2012 ▶); Chen *et al.* (2006 ▶).
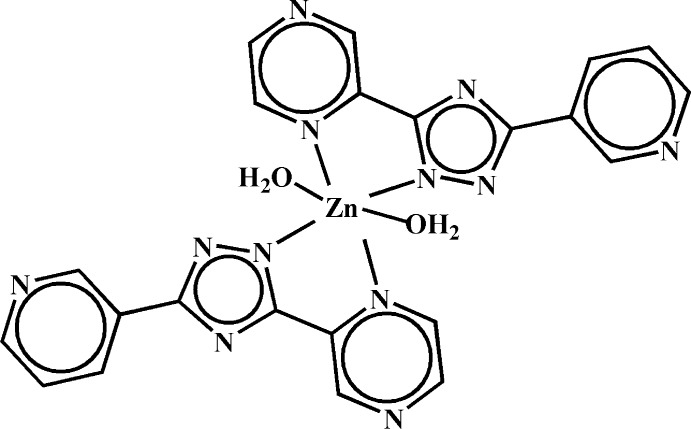


## Experimental   

### 

#### Crystal data  

[Zn(C_11_H_7_N_6_)_2_(H_2_O)_2_]
*M* *_r_* = 547.85
Monoclinic, 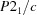

*a* = 8.600 (5) Å
*b* = 5.728 (3) Å
*c* = 22.288 (12) Å
β = 100.646 (6)°
*V* = 1079.0 (10) Å^3^
*Z* = 2
Mo *K*α radiation
μ = 1.19 mm^−1^
*T* = 296 K
0.18 × 0.15 × 0.13 mm

#### Data collection  

Bruker SMART 1000 CCD diffractometer
Absorption correction: multi-scan (*SADABS*; Bruker, 2001 ▶) *T*_min_ = 0.80, *T*_max_ = 0.86
11003 measured reflections
2493 independent reflections
2304 reflections with *I* > 2σ(*I*)
*R*_int_ = 0.083

#### Refinement  

*R*[*F* ^2^ > 2σ(*F* ^2^)] = 0.037
*wR*(*F* ^2^) = 0.110
*S* = 1.05
2493 reflections
175 parameters
3 restraints
H atoms treated by a mixture of independent and constrained refinement
Δρ_max_ = 0.44 e Å^−3^
Δρ_min_ = −0.59 e Å^−3^

### 

Data collection: *SMART* (Bruker, 2007 ▶); cell refinement: *SAINT* (Bruker, 2007 ▶); data reduction: *SAINT*; program(s) used to solve structure: *SHELXS97* (Sheldrick, 2008 ▶); program(s) used to refine structure: *SHELXL97* (Sheldrick, 2008 ▶); molecular graphics: *SHELXTL* (Sheldrick, 2008 ▶); software used to prepare material for publication: *SHELXTL*.

## Supplementary Material

Crystal structure: contains datablock(s) I, New_Global_Publ_Block. DOI: 10.1107/S1600536814004176/xu5772sup1.cif

Structure factors: contains datablock(s) I. DOI: 10.1107/S1600536814004176/xu5772Isup2.hkl

CCDC reference: 988429

Additional supporting information:  crystallographic information; 3D view; checkCIF report

## Figures and Tables

**Table 1:** Selected bond lengths (Å)

Zn1—O1	2.1054 (16)
Zn1—N1	2.1853 (18)
Zn1—N5	2.1733 (16)

**Table 2:** Hydrogen-bond geometry (Å, °)

*D*—H⋯*A*	*D*—H	H⋯*A*	*D*⋯*A*	*D*—H⋯*A*
O1—H1*A*⋯N6^i^	0.85 (2)	1.93 (2)	2.736 (2)	159 (2)
O1—H1*B*⋯N4^ii^	0.87 (2)	1.91 (2)	2.771 (2)	170 (2)
C1—H1⋯O1^iii^	0.93	2.41	3.266 (3)	153

Symmetry codes: (i) 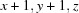; (ii) 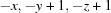; (iii) 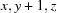.

**Fig. 1.**
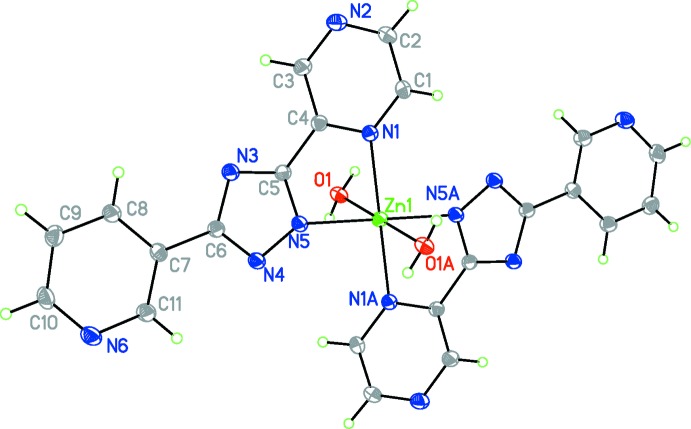
The structure of the title compound showing the atomic numbering and 30% probability displacement ellipsoids.

**Fig. 2.**
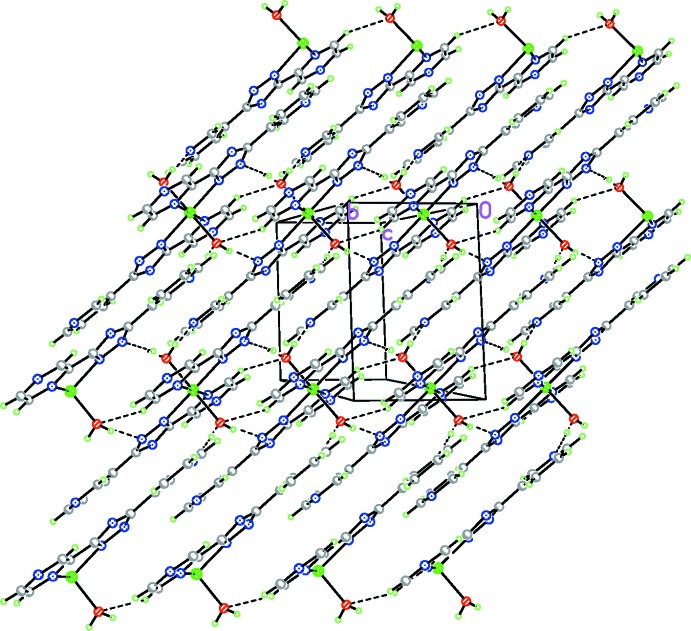
A packing diagram.

## supplementary crystallographic information

### 1. Comment

1,2,4-Triazole derivatives are important building blocks of many important compounds widely used in medicine, agriculture, industry, and coordination chemistry (Zhang *et al.*, 2012; Chen *et al.*, 2006). During the synthesis of polymeric complexes using 2-(5-(pyridin-3-yl)-4*H*-1,2,4-triazol-3-yl)pyrazine as building blocks and, to our surprise, the title monomeric Zn(II) complex was obtained.

The title compound is a crstallographically centrosymmetric mononuclear complex. The Zn^II^ cation is in a distaorted octahedral geometry (Fig. 1) and is coordinated by four N atoms from two 2-(5-(pyridin-3-yl)-1,2,4-triazolido-3-yl)pyrazine ligands and two coordinated water molecules. The observed Zn–O and Zn–N bond distances and bond angles reveal usual values. In the crystal, classic O–H···N hydrogen bonds, weak C—H···O hydrogen bond and π-π stacking between aromatic rings connect the molecules into the three dimensional supramolecular architecture [centroids between triazole and pyrazine rings and between triazole and pyridine rings being 3.623 (2) and 3.852 (2) Å, respectively].

### 2. Experimental

A mixture of 2-(5-(pyridin-3-yl)-4*H*-1,2,4-triazol-3-yl)pyrazine (0.0448 g, 0.2 mmol), Zn(CH_3_COO)_2_.2H_2_O (0.0220 g, 0.1 mmol), water (6 mL), *N*,*N*-dimethylformamide (2 mL) was stirred vigorously for 30 min and then sealed in a Teflon-lined stainless-steel autoclave. The autoclave was heated and maintained at 433 K for 3 d, and then cooled to room temperature at 5 K h^-1^ to obtain crystals suitable for X-ray analysis.

### 3. Refinement

All H-atoms were positioned geometrically and refined using a riding model with C–H = 0.93 Å, *U*_iso_(H) = 1.2 *U*_eq_(C) for aromatic hydrogen atoms. The H-atoms of O atoms were identified from a difference Fourier map and refined with O–H = 0.85 (2) Å, U_iso_(H) = 1.5U_eq_(O).

### Crystal data

**Table:**

[Zn(C_11_H_7_N_6_)_2_(H_2_O)_2_]	*F*(000) = 560
*M**_r_* = 547.85	*D*_x_ = 1.686 Mg m^−^^3^
Monoclinic, *P*2_1_/*c*	Mo *K*α radiation, λ = 0.71073 Å
Hall symbol: -P 2ybc	Cell parameters from 2880 reflections
*a* = 8.600 (5) Å	θ = 2.4–27.6°
*b* = 5.728 (3) Å	µ = 1.19 mm^−^^1^
*c* = 22.288 (12) Å	*T* = 296 K
β = 100.646 (6)°	Prism, colorless
*V* = 1079.0 (10) Å^3^	0.18 × 0.15 × 0.13 mm
*Z* = 2	

### Data collection

**Table:**

Bruker SMART 1000 CCD diffractometer	2493 independent reflections
Radiation source: fine-focus sealed tube	2304 reflections with *I* > 2σ(*I*)
Graphite monochromator	*R*_int_ = 0.083
phi and ω scans	θ_max_ = 27.6°, θ_min_ = 2.4°
Absorption correction: multi-scan (*SADABS*; Bruker, 2001)	*h* = −11→11
*T*_min_ = 0.80, *T*_max_ = 0.86	*k* = −7→7
11003 measured reflections	*l* = −29→28

### Refinement

**Table:**

Refinement on *F*^2^	Primary atom site location: structure-invariant direct methods
Least-squares matrix: full	Secondary atom site location: difference Fourier map
*R*[*F*^2^ > 2σ(*F*^2^)] = 0.037	Hydrogen site location: inferred from neighbouring sites
*wR*(*F*^2^) = 0.110	H atoms treated by a mixture of independent and constrained refinement
*S* = 1.05	*w* = 1/[σ^2^(*F*_o_^2^) + (0.0623*P*)^2^ + 0.2938*P*] where *P* = (*F*_o_^2^ + 2*F*_c_^2^)/3
2493 reflections	(Δ/σ)_max_ < 0.001
175 parameters	Δρ_max_ = 0.44 e Å^−^^3^
3 restraints	Δρ_min_ = −0.59 e Å^−^^3^

### Special details

**Table:**

Geometry. All e.s.d.'s (except the e.s.d. in the dihedral angle between two l.s. planes) are estimated using the full covariance matrix. The cell e.s.d.'s are taken into account individually in the estimation of e.s.d.'s in distances, angles and torsion angles; correlations between e.s.d.'s in cell parameters are only used when they are defined by crystal symmetry. An approximate (isotropic) treatment of cell e.s.d.'s is used for estimating e.s.d.'s involving l.s. planes.
Refinement. Refinement of *F*^2^ against ALL reflections. The weighted *R*-factor *wR* and goodness of fit *S* are based on *F*^2^, conventional *R*-factors *R* are based on *F*, with *F* set to zero for negative *F*^2^. The threshold expression of *F*^2^ > σ(*F*^2^) is used only for calculating *R*-factors(gt) *etc*. and is not relevant to the choice of reflections for refinement. *R*-factors based on *F*^2^ are statistically about twice as large as those based on *F*, and *R*- factors based on ALL data will be even larger.

### Fractional atomic coordinates and isotropic or equivalent isotropic displacement parameters (Å^2^)

**Table:**

	*x*	*y*	*z*	*U*_iso_*/*U*_eq_	
Zn1	0.0000	1.0000	0.5000	0.02623 (14)	
O1	0.17564 (15)	0.7938 (2)	0.47155 (6)	0.0298 (3)	
H1B	0.218 (3)	0.679 (4)	0.4940 (11)	0.045*	
H1A	0.252 (3)	0.871 (4)	0.4624 (11)	0.045*	
N1	−0.03718 (18)	1.1583 (3)	0.40926 (7)	0.0255 (3)	
N2	−0.0898 (2)	1.2803 (3)	0.28622 (8)	0.0364 (4)	
N3	−0.32425 (18)	0.7016 (3)	0.35570 (7)	0.0273 (3)	
N4	−0.27382 (18)	0.5864 (3)	0.45419 (7)	0.0271 (3)	
N5	−0.17912 (18)	0.7731 (3)	0.44860 (7)	0.0258 (3)	
N6	−0.6262 (2)	0.0459 (3)	0.41338 (9)	0.0357 (4)	
C1	0.0312 (2)	1.3446 (3)	0.38951 (9)	0.0302 (4)	
H1	0.0992	1.4355	0.4174	0.036*	
C2	0.0033 (3)	1.4058 (3)	0.32853 (9)	0.0339 (4)	
H2	0.0512	1.5393	0.3167	0.041*	
C3	−0.1585 (3)	1.0954 (4)	0.30615 (9)	0.0331 (4)	
H3	−0.2251	1.0039	0.2779	0.040*	
C4	−0.1352 (2)	1.0330 (3)	0.36728 (9)	0.0238 (4)	
C5	−0.2139 (2)	0.8348 (3)	0.38977 (8)	0.0242 (4)	
C6	−0.3581 (2)	0.5502 (3)	0.39810 (9)	0.0249 (4)	
C7	−0.4734 (2)	0.3611 (3)	0.38268 (8)	0.0261 (4)	
C8	−0.5332 (2)	0.3074 (4)	0.32153 (9)	0.0312 (4)	
H8	−0.5029	0.3951	0.2905	0.037*	
C9	−0.6372 (2)	0.1240 (4)	0.30748 (10)	0.0353 (4)	
H9	−0.6783	0.0865	0.2671	0.042*	
C10	−0.6790 (3)	−0.0028 (3)	0.35465 (12)	0.0362 (5)	
H10	−0.7475	−0.1285	0.3450	0.043*	
C11	−0.5265 (2)	0.2270 (4)	0.42666 (9)	0.0317 (4)	
H11	−0.4912	0.2645	0.4675	0.038*	

### Atomic displacement parameters (Å^2^)

**Table:**

	*U*^11^	*U*^22^	*U*^33^	*U*^12^	*U*^13^	*U*^23^
Zn1	0.0291 (2)	0.0287 (2)	0.0197 (2)	−0.00523 (11)	0.00119 (13)	0.00147 (10)
O1	0.0282 (7)	0.0286 (7)	0.0331 (7)	−0.0035 (5)	0.0064 (5)	0.0056 (5)
N1	0.0268 (7)	0.0255 (7)	0.0238 (7)	−0.0033 (6)	0.0040 (6)	0.0002 (5)
N2	0.0442 (10)	0.0369 (9)	0.0274 (8)	−0.0051 (8)	0.0044 (7)	0.0068 (7)
N3	0.0269 (7)	0.0293 (8)	0.0249 (7)	−0.0060 (6)	0.0023 (6)	0.0017 (6)
N4	0.0266 (7)	0.0279 (8)	0.0264 (8)	−0.0056 (6)	0.0042 (6)	0.0020 (6)
N5	0.0273 (7)	0.0253 (7)	0.0240 (7)	−0.0057 (6)	0.0030 (6)	0.0013 (6)
N6	0.0303 (9)	0.0395 (9)	0.0382 (10)	−0.0096 (7)	0.0089 (8)	0.0040 (8)
C1	0.0326 (9)	0.0266 (9)	0.0314 (9)	−0.0077 (7)	0.0059 (7)	−0.0027 (7)
C2	0.0414 (11)	0.0271 (9)	0.0344 (10)	−0.0045 (8)	0.0105 (8)	0.0050 (8)
C3	0.0386 (10)	0.0359 (10)	0.0234 (9)	−0.0081 (8)	0.0018 (7)	0.0009 (8)
C4	0.0229 (8)	0.0257 (8)	0.0227 (9)	−0.0007 (6)	0.0036 (7)	0.0005 (6)
C5	0.0237 (8)	0.0266 (9)	0.0222 (8)	−0.0032 (7)	0.0036 (6)	0.0002 (6)
C6	0.0212 (8)	0.0289 (8)	0.0246 (9)	−0.0035 (7)	0.0041 (7)	0.0005 (7)
C7	0.0203 (8)	0.0277 (8)	0.0300 (9)	−0.0035 (7)	0.0040 (7)	−0.0002 (7)
C8	0.0295 (9)	0.0353 (10)	0.0281 (9)	−0.0078 (8)	0.0040 (7)	0.0013 (8)
C9	0.0318 (10)	0.0409 (11)	0.0324 (10)	−0.0073 (8)	0.0036 (8)	−0.0064 (8)
C10	0.0306 (11)	0.0336 (12)	0.0442 (14)	−0.0114 (7)	0.0063 (10)	−0.0056 (8)
C11	0.0250 (8)	0.0396 (10)	0.0304 (9)	−0.0077 (8)	0.0048 (7)	0.0014 (8)

### Geometric parameters (Å, º)

**Table:**

Zn1—O1^i^	2.1054 (16)	N6—C10	1.334 (3)
Zn1—O1	2.1054 (16)	N6—C11	1.344 (3)
Zn1—N1	2.1853 (18)	C1—C2	1.381 (3)
Zn1—N1^i^	2.1853 (18)	C1—H1	0.9300
Zn1—N5	2.1733 (16)	C2—H2	0.9300
Zn1—N5^i^	2.1733 (16)	C3—C4	1.387 (3)
O1—H1B	0.867 (16)	C3—H3	0.9300
O1—H1A	0.848 (16)	C4—C5	1.457 (2)
N1—C1	1.332 (2)	C6—C7	1.466 (3)
N1—C4	1.345 (2)	C7—C11	1.387 (3)
N2—C3	1.329 (3)	C7—C8	1.399 (3)
N2—C2	1.330 (3)	C8—C9	1.377 (3)
N3—C5	1.339 (2)	C8—H8	0.9300
N3—C6	1.353 (2)	C9—C10	1.379 (3)
N4—C6	1.340 (2)	C9—H9	0.9300
N4—N5	1.364 (2)	C10—H10	0.9300
N5—C5	1.338 (2)	C11—H11	0.9300
			
O1^i^—Zn1—O1	180.00 (7)	N2—C2—C1	122.23 (18)
O1^i^—Zn1—N5	90.93 (7)	N2—C2—H2	118.9
O1—Zn1—N5	89.07 (7)	C1—C2—H2	118.9
O1^i^—Zn1—N5^i^	89.07 (7)	N2—C3—C4	122.76 (18)
O1—Zn1—N5^i^	90.93 (7)	N2—C3—H3	118.6
N5—Zn1—N5^i^	180.0	C4—C3—H3	118.6
O1^i^—Zn1—N1	93.18 (6)	N1—C4—C3	120.28 (17)
O1—Zn1—N1	86.82 (6)	N1—C4—C5	116.58 (17)
N5—Zn1—N1	78.00 (6)	C3—C4—C5	123.13 (17)
N5^i^—Zn1—N1	102.00 (6)	N5—C5—N3	114.31 (16)
O1^i^—Zn1—N1^i^	86.82 (6)	N5—C5—C4	120.63 (16)
O1—Zn1—N1^i^	93.18 (6)	N3—C5—C4	125.06 (16)
N5—Zn1—N1^i^	102.00 (6)	N4—C6—N3	113.79 (17)
N5^i^—Zn1—N1^i^	78.00 (6)	N4—C6—C7	123.96 (17)
N1—Zn1—N1^i^	180.000 (1)	N3—C6—C7	122.22 (17)
Zn1—O1—H1B	120.2 (18)	C11—C7—C8	117.17 (17)
Zn1—O1—H1A	114.1 (18)	C11—C7—C6	122.69 (17)
H1B—O1—H1A	106 (2)	C8—C7—C6	120.14 (17)
C1—N1—C4	117.11 (16)	C9—C8—C7	119.71 (18)
C1—N1—Zn1	129.81 (13)	C9—C8—H8	120.1
C4—N1—Zn1	112.83 (12)	C7—C8—H8	120.1
C3—N2—C2	116.14 (17)	C8—C9—C10	118.57 (19)
C5—N3—C6	100.99 (15)	C8—C9—H9	120.7
C6—N4—N5	105.37 (15)	C10—C9—H9	120.7
C5—N5—N4	105.55 (14)	N6—C10—C9	123.25 (19)
C5—N5—Zn1	111.52 (11)	N6—C10—H10	118.4
N4—N5—Zn1	142.93 (12)	C9—C10—H10	118.4
C10—N6—C11	117.76 (19)	N6—C11—C7	123.48 (19)
N1—C1—C2	121.43 (18)	N6—C11—H11	118.3
N1—C1—H1	119.3	C7—C11—H11	118.3
C2—C1—H1	119.3		

Symmetry code: (i) −*x*, −*y*+2, −*z*+1.

### Hydrogen-bond geometry (Å, º)

**Table:**

*D*—H···*A*	*D*—H	H···*A*	*D*···*A*	*D*—H···*A*
O1—H1*A*···N6^ii^	0.85 (2)	1.93 (2)	2.736 (2)	159 (2)
O1—H1*B*···N4^iii^	0.87 (2)	1.91 (2)	2.771 (2)	170 (2)
C1—H1···O1^iv^	0.93	2.41	3.266 (3)	153

Symmetry codes: (ii) *x*+1, *y*+1, *z*; (iii) −*x*, −*y*+1, −*z*+1; (iv) *x*, *y*+1, *z*.
